# What Time Periods of the Day Are Concerning for Parents of Children with Attention Deficit Hyperactivity Disorder?

**DOI:** 10.1371/journal.pone.0079806

**Published:** 2013-11-05

**Authors:** Masahide Usami, Takashi Okada, Daimei Sasayama, Yoshitaka Iwadare, Kyota Watanabe, Hirokage Ushijima, Masaki Kodaira, Nobuhiro Sugiyama, Tetsuji Sawa, Kazuhiko Saito

**Affiliations:** 1 Department of Child and Adolescent Psychiatry, National Center for Global Health and Medicine, Kohnodai Hospital, Ichikawa, Japan; 2 Department of Child and Adolescent Psychiatry, Nagoya University Graduate School of Medicine, Nagoya, Japan; 3 Department of Child and Adolescent Mental Health, Imperial Gift Foundation, Aiiku Maternal and Child Health Center, Aiiku Hospital, Minato-ku, Japan; 4 Department of Neuropsychiatry, Shinshu University School of Medicine, Matsumoto, Japan; 5 Department of Developmental Psychiatry, Graduate School of Medical Science, Kitasato University, Sagamihara, Japan; Hangzhou Normal University, China

## Abstract

**Background/Aim:**

The questionnaire-children with difficulties (QCD) is a parent-assessed questionnaire designed to evaluate a child’s difficulties in functioning during specific time periods of the day. In this study, the QCD was applied to determine the time periods of the day that are concerning for the parents of children with attention deficit hyperactivity disorder (ADHD). The results were compared with those for a community sample.

**Methods:**

Elementary and junior high school students with ADHD (243 boys, 55 girls) and a community sample of children (518 boys, 618 girls) were enrolled in this study. Their behaviors were assessed by the QCD, the ADHD-rating scale (ADHD-RS), and the Oppositional Defiant Behavior Inventory (ODBI). The effects of gender (boy/girl) and diagnosis (ADHD/community sample) on the total QCD score were analyzed across each school grade (elementary/junior high school). Correlation coefficients between QCD and ADHD-RS/ODBI scores were analyzed.

**Results:**

The QCD score for the ADHD group was significantly lower than that for the community sample (P < 0.001). There were significantly strong correlations between “evening” and ADHD-RS and ODBI scores for all children with ADHD (r > 0.41, P < 0.001) and between “night” and inattention and oppositional symptoms for the girls with ADHD (r > 0.40, P < 0.001).

**Conclusions:**

Parents reported that children with ADHD faced greater difficulties in completing basic daily activities compared with the community controls, particularly in the evening. Furthermore, these difficulties were related to the severity of ADHD symptoms. The parents’ perceptions depended on the gender, ADHD and oppositional symptoms, and the time period of the day. This study determined that children with ADHD face greater difficulties in daily functioning compared with community sample children, that these difficulties are time-dependent, and that these difficulties were particularly experienced in the evening.

## Introduction

Attention deficit hyperactivity disorder (ADHD) is a neurodevelopmental disorder with core symptoms of hyperactivity, impulsiveness, and inattention. These symptoms are continuously recognized throughout the daytime from childhood to adulthood [1-4]. It has been suggested that children with ADHD face difficulties in various aspects of life, such as family relationships, school life, friendships, academic achievement, emotions in adulthood, and work, while severity is associated with the core symptoms of ADHD [1-9]. Some studies have indicated a relationship between ADHD symptoms in children and family burden [10-13].

Evaluation of a child’s functioning across various periods of the day and a trial of appropriate treatment are important from the perspective of the long-term prognosis of ADHD in children and family burden [14]. Certain periods of the day are important for children to acquire social skills and engage in family life through relationships with their friends and parents [5,6,15]. 

The Questionnaire-Children with Difficulties (QCD) has been widely used to evaluate a parent’s perceptions of their child’s daily behaviors during specific periods of the day, such as morning, during school, after school, evening, and night [15-17]. The QCD is also practical for sharing information among caretakers because it enables the evaluation of life function at each period of the day compared with the ADHD Rating Scale-IV published in 2008 [5,15,17-20]. The QCD is superior in terms of clarifying difficulties in daily functioning encountered by children with ADHD throughout the day. Only a short period of time is required to complete the QCD compared with the Child Behavior Checklist (CBCL) [5,6]. Usami and colleagues standardized the QCD [17].

However, no study has used the QCD to examine difficulties in daily functioning in children with ADHD compared with those in typically developing children. The use of the QCD in children with ADHD enables one to elucidate ADHD symptom-associated difficulties in daily functioning during different time periods of the day.

In this study, we aimed to use the QCD to evaluate difficulties in daily functioning associated with ADHD during different time periods of the day. A major hypothesis of this study was that the QCD scores of children with ADHD exhibit significantly strong correlations with ADHD symptoms reported during all time periods of the day. Because boys have more severe ADHD symptoms compared with girls as per the ADHD-rating scale (ADHD-RS) score, a minor hypothesis of this study was that boys with ADHD exhibit significantly lower QCD scores compared with girls with ADHD. This hypothesis indicated that the family burden was more severe on boys than on girls with ADHD.

ADHD is often comorbid with other neuropsychiatric disorders [5,21], one of the more frequent ones being oppositional defiant disorder (ODD) [5,21,22]. As such, ODD comorbid with ADHD, rather than pure ADHD, may be associated with QCD scores. To examine this possibility, we examined ODD symptoms as well as ADHD symptoms in our study subjects. 

## Methods

### Study Design and Setting

This study was a survey administered as a paper questionnaire. The Kohnodai Hospital is located in Ichikawa City in the eastern part of the Tokyo metropolitan area. No real data was available regarding the socioeconomic status of the citizens of Ichikawa City. The city has fully developed into a residential area and center for education with an estimated population of 468,275 (as of February 2013), making it the fourth largest city in the prefecture.

### Recruitment and Participants

The ethical committee of the National Center for Global Health and Medicine approved this study. 

Using the QCD, we performed a case–control study of the relationship between ADHD symptoms and parents’ perceptions. There were two subject groups of elementary school and junior high school students in this study: an ADHD group and a community control sample of typically developing children. 

### ADHD Group

All 298 individuals in the ADHD group [243 boys, 55 girls; mean age, 9.6 ± 2.8 years; range, 6–15 years] were examined and diagnosed with the combined subtype of ADHD according to the DSM-IV-TR by psychiatrists with specialization in child and adolescent psychiatry at the Department of Child and Adolescent Psychiatry, Kohnodai Hospital, National Center for Global Health and Medicine between September 4, 2008 and March 31, 2012. Seventy-six ADHD patients diagnosed with the inattentive type and 34 diagnosed with the hyperactivity–impulsivity type were excluded, as were patients with coexisting mental disorders such as intellectual disability, pervasive developmental disorders, eating disorders, mood disorders, oppositional defiant disorder, and conduct disorder. All children in the ADHD group were outpatients and drug-naive. To minimize confounding factors, we selected only the combined subtype of ADHD without coexisting disorders.

The ADHD group completed the QCD, ADHD-RS, and Oppositional Defiant Behavior Inventory (ODBI) as routine screening tools during the initial examination at the child and adolescent psychiatry outpatient clinic of the Kohnodai Hospital. Researchers as well as all staff members of our hospital had access to the ADHD-RS, ODBI, and other clinical data from the patient’s medical records. Data pertaining to the QCD, ADHD-RS, and ODBI for the ADHD group were obtained from the patients’ clinical records. Along with documented informed consent from each patient, the guardians also provided informed consent for participation. The ethical committee of the National Center for Global Health and Medicine approved this consent procedure.　

### Community Sample

The community sample was surveyed in cooperation with the Board of Education in Ichikawa City [17]. The QCD, ADHD-RS, and ODBI were distributed by teachers to the parents of 10,242 randomly selected children at 11 public elementary schools (7,111 children) and 5 junior high schools (3,131 children) in Ichikawa City, Chiba Prefecture. 

First, the survey method was explained to the principals of all schools by the Education Committee of Ichikawa City. Next, the teachers distributed a letter written by the Education Committee to explain the survey to all children and their parents. The letter clearly stated that completion of the consent form by a parent would be considered provision of consent to the survey from both the parents and students. The letter also specified that the results would be published as a medical paper in a format that individual is not specified. This consent procedure was approved by the ethical committee of the National Center for Global Health and Medicine.

QCD data for the community sample was obtained from 1,803 parents who provided informed consent to the mailed survey. Of these, 1,136 questionnaires that were completely filled out were analyzed. We discussed this QCD and ADHD-RS data in a previous study [17]. Children in the community sample were not evaluated for mental disorders such as ADHD, pervasive developmental disorder, intellectual disability, or mood disorders. 

### Measures

#### QCD

The QCD comprises 20 questions related to activities that occur during specific periods of the day: questions 1–4, early morning/before going to school; questions 5–7, during school; questions 8–10, after school; questions 11–14, evening; questions 15–18, at night; and questions 19 and 20, overall behavior (Appendix). Each question is scored on the basis of the following four grades: 0 = completely disagree, 1 = somewhat (partially) agree, 2 = mostly agree, and 3 = completely agree. The QCD comprises the total score and six subscales (early morning/before going to school, school, after school, evening, night, overall behavior). Higher scores indicate higher life function and less difficulty in daily activities during specific periods of the day. The questionnaire is composed of practical and easy-to-understand questions regarding basic daily activities such as face washing, brushing teeth, and dressing. These subscales of the QCD were revised by Yamashita to match the daily life of Japanese children without using factor analysis [5,14]. Target children whose parents could answer the QCD were identified to be elementary and junior high school students. Usami and colleagues standardized the QCD [17].

#### ADHD-RS

The ADHD-RS comprises 18 questions about hyperactivity, impulsiveness, and inattention. Four possible responses are recorded for each question: “generally none,” “usually none,” “usually exists,” or “always exists”, scored as 0, 1, 2, or 3 points, respectively. The total score is used for evaluation. Higher scores indicate multiple and more severe symptoms. The ADHD-RS is widely used in Japanese clinical practice and clinical research [23]. 

#### ODBI

Harada and colleagues developed and standardized theODBI [20]. The ODBI comprises 18 items to be answered by the caregiver. Four possible responses are recorded for each question: “generally none,” “usually none,” “usually exists,” or “always exists”, scored as 0, 1, 2, or 3 points, respectively, and the total score is used for evaluation. The cutoff score was 20 points, and subjects whose ODBI score was over 20 points were considered to be in the high ODBI subgroup. A higher score indicated a greater severity of oppositional defiant behavior. The ODBI is widely used in Japanese clinical practice and clinical research [23].

### Statistical Analysis

Scores for the questions in each of the 6 subcategories and the total QCD score were all determined separately. Six subscores and the total QCD score were statistically compared between the ADHD group and the community sample using an unpaired t-test. These effect sizes were calculated.

The effects of gender and diagnosis (ADHD group, community sample) on the total QCD score were analyzed using two-way analysis of variance. The significance threshold was defined as P < 0.0071, which corresponds to the Bonferroni-adjusted P < 0.05 corrected for the number of subscales (N = 7). Spearman’s correlation coefficient was calculated to examine whether the total QCD score and subscores correlated with the hyperactivity/impulsiveness score and the attention deficit score on the ADHD-RS and the ODBI score. The significance threshold for correlations of QCD subscale scores with ADHD-RS and ODBI scores was defined as P < 0.0012, which corresponds to the Bonferroni-adjusted P < 0.05 corrected for the number of tests performed (N = 42, i.e., 6 tests for each of the 7 subscale scores)

All statistical tests were two-tailed, and P < 0.0071 or P < 0.0012 indicated statistical significance. Analyses were performed using PASW Statistics 18.0 statistical software (IBM Japan Inc.). The raw data used for the analyses described in this manuscript are available upon request.

## Results

### Distributions of the QCD Scores

QCDs that were filled out completely were collected from the parents of the children in the ADHD group and the community sample (Table 1). The 6 QCD subscores and the total score were significantly lower in the ADHD group than in the community sample (P < 0.001). Effect sizes were greater than 0.983.

**Table 1:** Clinical data for the ADHD group and the community sample.

			ADHD group	Community sample	P value	Effect size
Number (boy/girl)			298 (243/55)	1136 (518/618)	-	-
Age (mean ± SD)			9.79 ± 2.65	9.83 ± 2.50	N.S.	0.003
QCD score (mean ± SD)	Morning		5.04 ± 3.11	8.58 ± 2.56	<0.0001	1.168
	School		5.10 ± 2.07	7.97 ± 1.42	<0.0001	1.491
	After school		5.35 ± 2.13	7.71 ± 1.51	<0.0001	1.245
	Evening		5.48 ± 2.69	10.17 ± 1.93	<0.0001	1.656
	Night		6.51 ± 2.32	8.19 ± 1.36	<0.0001	0.983
	Overall behavior		2.21 ± 1.59	5.08 ± 1.07	<0.0001	1.726
	Total score		29.70 ± 9.86	47.70 ± 7.15	<0.0001	1.698
ADHD-RS (mean ± SD)	Hyperactivity		18.02 ± 6.10	2.50 ± 3.22	<0.0001	2.098
	Inattention		28.08 ± 10.69	3.20 ± 4.27	<0.0001	2.118
ODBI (mean ± SD)	Total score		29.27 ± 13.22	9.51 ± 6.49	<0.0001	1.714

### QCD Scores by Diagnosis and Gender

The average total QCD score and QCD subscores were compared within each gender (Table 2). The total score and subscores were significantly lower for the ADHD group than for the community sample within each gender (P < 0.001). 

**Table 2:** Total QCD score and subscores.

QCD	Boy				Girl						
	Mean	SD	N		Mean	SD	N			f	p
Morning									Diag × Gender	8.882	0.0029
Community sample	8.50	2.55	518		8.64	2.56	618		Gender	5.421	N.S.
ADHD group	5.25	3.14	243		4.11	2.77	55		Diag	328.1	<0.0001
School									Diag × Gender	1.913	N.S.
Community sample	7.82	1.49	518		8.10	1.35	618		Gender	0.6887	N.S.
ADHD group	5.12	2.10	243		5.05	1.94	55		Diag	516.4	<0.0001
After school									Diag × Gender	0.7492	N.S.
Community sample	7.53	1.56	518		7.85	1.45	618		Gender	2.381	N.S.
ADHD group	5.33	2.17	243		5.42	1.98	55		Diag	303.6	<0.0001
Evening									Diag × Gender	5.773	N.S.
Community sample	9.95	2.03	518		10.36	1.81	618		Gender	0.00088	N.S.
ADHD group	5.56	2.71	243		5.16	2.58	55		Diag	809.2	<0.0001
Night									Diag × Gender	0.6646	N.S.
Community sample	8.23	1.30	518		8.15	1.40	618		Gender	2.063	N.S.
ADHD group	6.56	2.32	243		6.27	2.30	55		Diag	189.9	<0.0001
Overall behavior									Diag × Gender	10.07	0.0015
Community sample	5.03	1.10	518		5.12	1.05	618		Gender	5.005	N.S.
ADHD group	2.32	1.59	243		1.80	1.53	55		Diag	984.2	<0.0001
Total score									Diag × Gender	7.679	0.0057
Community sample	47.07	7.33	518		48.22	6.96	618		Gender	0.8631	N.S.
ADHD group	30.13	9.95	243		27.82	9.26	55		Diag	894.4	<0.0001

### Correlation with Hyperactivity, Inattention, and ODBI

Correlations of the total QCD score and subscores with the hyperactivity/impulsiveness score and the inattention score of the ADHD-RS or the total ODBI score are shown in Table 3. All correlations were significant. The correlations of “School” and “After School” subscores with ADHD symptoms were under 0.31 in both the ADHD group and the community sample. Correlations of “Evening” and ADHD-RS with ODBI scores were significantly strong (r > 0.41, P < 0.001) for all children with ADHD. Furthermore, correlations between “Night” and inattention and oppositional symptoms were significantly strong (r > 0.40, P < 0.001) for the girls with ADHD. 

**Table 3:** Correlation of the QCD score with the ADHD-RS and ODBI scores.

QCD	Correlation with					
	Hyperactivity/impulsiveness		Inattention		ODBI	
	Boy	Girl	Boy	Girl	Boy	Girl
Morning						
Community sample	−0.33	−0.46	−0.32	−0.45	−0.33	−0.36
ADHD group	−0.35	−0.36	−0.30	−0.35	−0.33	−0.39
School						
Community sample	−0.25	−0.17	−0.24	−0.16	−0.21	−0.16
ADHD group	−0.23	−0.27	−0.23	−0.31	−0.25	−0.41
After school						
Community sample	−0.29	−0.29	−0.28	−0.28	−0.21	−0.21
ADHD group	−0.29	−0.11	−0.29	−0.08	−0.19	−0.37
Evening						
Community sample	−0.46	−0.43	−0.46	−0.42	−0.46	−0.47
ADHD group	−0.43	−0.48	−0.44	−0.58	−0.41	−0.49
Night						
Community sample	−0.15	−0.23	−0.16	−0.24	−0.19	−0.19
ADHD group	−0.24	−0.25	−0.23	−0.41	−0.26	−0.49
Overall behavior						
Community sample	−0.33	−0.32	−0.33	−0.34	−0.40	−0.33
ADHD group	−0.31	−0.24	−0.37	−0.28	−0.55	−0.53
Total score						
Community sample	−0.45	−0.49	−0.45	−0.49	−0.43	−0.43
ADHD group	−0.44	−0.36	−0.43	−0.44	−0.47	−0.64

All P values are <0.0014

Bold: correlation coefficient< −0.40, All P < 0.0012

## Discussion

This study is the first, as per our knowledge, to examine QCD scores in relation to ADHD-RS and OBDI scores in a community sample of children as well as children with ADHD during various time periods of the day. Regardless of the gender, we found that the QCD scores were significantly lower for the ADHD group than for the community sample during every time period; furthermore, they were associated with ADHD-RS and ODBI scores. These results indicate that ADHD children face difficulties in daily functioning, which are associated with ADHD and ODBI symptoms. Correlations were comparatively higher in the evening, suggesting that the QCD reflects difficulties at home that may be associated with family burden. When parents answered the QCD, they reported that they were more concerned to care for their children with ADHD in the evening. Particularly after they returned home, children with ADHD did their homework with greater difficulties and did not enjoy family time because of constant quarreling with others. Therefore, the parents felt uncomfortable in spending time with the child while engaging in evening activities. Considering the results, the hypothesis that the family burden was more severe on boys with ADHD was rejected. However, functional problems of girls with ADHD are more strongly correlated with their ADHD and oppositional symptoms during the night-time compared with those of boys. The results indicated that the family burden for children with ADHD is related to gender, ADHD symptoms, oppositional symptoms and the time period. 

 These findings have implications for treatment planning. The children in the study were not yet on medication. Nevertheless, comprehensive treatment consisting of parent training based on learning principles and behavioral therapy, psychotherapy, social therapy, and medication is recommended in the guidelines for the treatment of ADHD in Japan[14]. Medication is usually attempted after other behavioral approaches, such as parent training and psychotherapy. However, only two drugs, long-acting methylphenidate and the nonstimulant atomoxetine, are currently approved for the treatment of ADHD in Japan[14]. Stimulant medication can be helpful with daytime symptoms, but it is typically not an option in the evening/ night because of the effects on sleep. Even in children with ADHD who are not on medication, such as those in this study, sleep problems are very common in ADHD and impact quality of life. In some cases further assessment of sleep is required (such as polysomnography in the case of possible apnea), but behavioral strategies will prove helpful in most cases. Treatment planning must take into account the difficulties faced by children with ADHD in their functioning during the evening and at night, including sleep problems. Useful behavioral management strategies may include checklists, rewards/ reinforcements, parent training, and sleep hygiene approaches. 

This study has some limitations that need to be considered. Children in the ADHD group were diagnosed with the combined subtype of ADHD. Furthermore, we have excluded children with ADHD and actual comorbid diagnoses. This study did not evaluate difficulties in daily functioning that are related to the inattention subtype or the hyperactivity–impulsivity subtype of ADHD. This result showed the relationship between oppositional behavior as reported in a questionnaire and ADHD symptoms, but the ability to examine this is constrained by excluding children diagnosed with ADHD and ODD.

The subjects were recruited from the general population of one district and a population of children with ADHD in one national center in Japan. That national center was located in the same district; however, outpatients from the center were children from different districts. 

Therefore, the present findings should be carefully generalized to populations at large. Also, children in the community sample did not undergo psychiatric evaluation; therefore, the presence of mental disorders could not be determined in these children. Such factors must be taken into account when determining reference values and cutoff scores. Future studies should include participants from different districts to minimize the effects of district-specific factors.

 This study demonstrated that the family burden for girls with ADHD during the night time was more severe and ADHD and sleep problems are highly interrelated, both bing impacted by medications [24]. However, medications were not administered to children with ADHD in this study, and none of them answered any questionnaire regarding night-time behaviors and sleep problems. Therefore, this study is insufficient because the survey was conducted for night-time behavior and sleep problems for children with ADHD and between boys and girls with ADHD. Examinations by a child psychiatrist using activity monitoring devices, and night-time polysomnography are necessary for accurately assessing sleep problems in children with ADHD. 

In summary, this study determined that children with ADHD face more difficulties in daily functioning compared with community sample children. These difficulties are dependent on gender and time period, and parents experience them particularly in the evening. Use of the QCD enables clinicians to inform parents about specific time periods during which their children experience ADHD-related difficulties and oppositional defiant symptoms in their daily life. 

## Supporting Information

Appendix S1 Questionnaire-Children with Difficulties (English version*). To prevent misinterpretation and biases, two Japanese psychiatrists with a good command of English, who understood the background and objectives of the evaluation scale, independently carried out forward translation of the QCD into English. Then, the two translators discussed and integrated the two translated versions into one. Another psychiatrist did back translation to Japanese, the original language. The back-translated version was examined by the author of the original version and it was confirmed that the intent of the author was accurately translated. After the final proofreading, construction of the QCD English version was completed.
(DOCX)
