# Supplementary material for: What Time Periods of the Day Are Concerning for Parents of Children with Attention Deficit Hyperactivity Disorder?
Source: PLoS One. 2013 Nov 5;8(11):e79806. doi: 10.1371/journal.pone.0079806 (PMC3818216; doi:10.1371/journal.pone.0079806)
Supplement: Appendix S1 — Questionnaire-Children with Difficulties (English version*). To prevent misinterpretation and biases, two Japanese psychiatrists with a good command of English, who understood the background and objectives of the evaluation scale, independently carried out forward translation of the QCD into English. Then, the two translators discussed and integrated the two translated versions into one. Another psychiatrist did back translation to Japanese, the original language. The back-translated version was examined by the author of the original version and it was confirmed that the intent of the author was accurately translated. After the final proofreading, construction of the QCD English version was completed. (DOCX) [file pone.0079806.s001.docx]

Appendix 1. Questionnaire-Children with Difficulties (English version*)

| **Early morning/before going to school** | | | | | |
| --- | --- | --- | --- | --- | --- |
| 1. Can your child promptly get out of his/her bed? | | | | | |
| 2. Can your child promptly groom himself/herself (for example, washing face, brushing teeth and getting dressed)? | | | | | |
| 3. Can your child behave in an age-appropriate manner at breakfast? | | | | | |
| 4. Can your child spend his/her time before going to school in the morning without getting into trouble or having quarrels with his/her parents or siblings? | | | | | |
| **School** |  |  |  |  |  |
| 5. Does your child like going to school? | | | | | |
| 6. Can your child behave in class as other children do? | | | | | |
| 7. Does your child have friends who accept him/her at school? | | | | | |
| **After school** | | |  |  |  |
| 8. Can your child discuss events that happened at school with his/her parents/guardian? | | | | | |
| 9. Does your child have friends of his/her own age? | | | | | |
| 10. Can your child confidently participate in extracurricular activities, such as sports, with children of his/her own age? | | | | | |
| **Evening** |  |  |  |  |  |
| 11. Can your child do his/her homework at home without difficulties? | | | | | |
| 12. After everyone returns home (including parents/guardians), can your child enjoy family time without constantly quarreling with others? | | | | | |
| 13. Can your child converse in a calm manner during dinnertime conversations? | | | | | |
| 14. Do the parents feel comfortable being together with the child when engaging in activities (for example, going out or shopping)? | | | | | |
| **Night** |  |  |  |  |  |
| 15. Adolescent child (12 years or older): | | | | | |
| Can your child engage in activities at night with friends of his/her own age? These activities may include playing, studying, going to cram school, taking private lessons (for example, playing a musical instrument and/or calligraphy), and playing sports. | | | | | |
| 16. Younger children (younger than 12 years): | | | | | |
| Can your child follow instructions at night (for example, brushing teeth, changing clothes)? | | | | | |
| 17. Can your child go to sleep without any difficulties? | | | | | |
| 18. Is your child sleeping without waking up during the night? | | | | | |
| **Overall behavior** |  |  |  |  |  |
| 19. Does your child have self-confidence? Is your child socially accepted by others (such as belonging to a group of his/her friends), and emotionally stable? | | | | | |
| 20. Does your child have more days in the week, where he/she is able to spend the day without facing confusion, getting into quarrels or displaying rebellious behavior? | | | | | |
| To prevent misinterpretation and biases, two Japanese psychiatrists with a good command of English, who understood the background and objectives of the evaluation scale, independently carried out forward translation of the QCD into English. Then, the two translators discussed and integrated the two translated versions into one. Another psychiatrist did back translation to Japanese, the original language. The back-translated version was examined by the author of the original version and it was confirmed that the intent of the author was accurately translated. After the final proofreading, construction of the QCD English version was completed. | | | | | |
